# 649. Expansion Of A Low-Cost, Open-Source, Saliva-Based PCR Test For The Detection Of Mpox Virus

**DOI:** 10.1093/ofid/ofad500.712

**Published:** 2023-11-27

**Authors:** Russell Thomas, Devyn Yolda-Carr, Sydney A Steel, Emily R Tobik, Theresa Zepeda, Maurice Brownlee, Shyam Saladi, James Parkin, Katherine Fajardo, Orchid M Allicock, Anne L Wyllie

**Affiliations:** Yale University, New Haven, Connecticut; Yale School of Public Health, New Haven, Connecticut; Yale School of Public Health, New Haven, Connecticut; Yale University, New Haven, Connecticut; Neelyx Labs, Streamwood, Illinois; Baal Perazim Wellness & Health Services Inc., Chicago, Illinois; Neelyx Labs, Streamwood, Illinois; Neelyx Labs, Streamwood, Illinois; Yale University, New Haven, Connecticut; Yale School of Public Health, New Haven, Connecticut; Yale School of Public Health, New Haven, Connecticut

## Abstract

**Background:**

Clinical diagnosis of mpox (monkeypox) virus relies on lesion swabs. Many patients experience prodromal symptoms prior to lesion-onset. Earlier detection could bolster outbreak response by mitigating transmission and facilitating access to antiviral treatments. With a growing number of studies reporting mpox detection in saliva, we adapted our saliva-based, extraction-free PCR test developed for SARS-CoV-2, for the detection of mpox virus.

**Methods:**

We first compared five PCR assays for their detection of mpox DNA extracted from 30 saliva specimens collected into Spectrum SDNA-1000 tubes. Next, we investigated the stability of mpox detection in five raw, unsupplemented saliva samples diluted 1:5 in mpox-negative saliva, after storage at 4°C, ∼19°C (room temp.), 30°C, and 40°C for 72 hours. We also examined the stability of virus detection in three samples as per the FDA guidance for shipping of samples for testing. Additionally, we performed amplicon sequencing on 10 saliva samples and assessed concordance of the PCR assays against mpox virus sequences.

**Results:**

The Logix Smart™ Mpox (2-Gene) RUO (Co-Diagnostics, Inc.) performed better at detection. Despite identifying three different substitutions in the CDC’s Monkeypox Virus Generic Real-Time PCR Test’s primers, results from ANOVA did not suggest a mean difference in cycle threshold values between assays. Detection following storage at 4°C, ∼19°C, and 30°C remained relatively stable for 24-48 hours but this declined by 72 hours. At 40°C, detection was stable at 24 hours but declined by 48 hours. Detection following simulated summer and winter shipping temperature profiles also remained stable.

**Conclusion:**

Findings of this pilot investigation support a flexible, extraction-free, saliva-based PCR test as a promising approach for the low-cost detection of mpox virus. With stability observed for 24-48 hours as well as over simulated shipping temperatures, saliva-based sampling and simplified testing could reduce mpox diagnostic costs, increase access to testing and address hurdles in low- and middle-income countries. Future studies should assess the temporal dynamics of mpox virus in saliva.

**Disclosures:**

**Shyam Saladi, n/a**, Neelyx Labs: Ownership Interest|SalivaDirect Inc: Board Member **James Parkin, PhD**, Neelyx Labs: employee **Anne L. Wyllie, PhD**, Co-Diagnostics: Board Member|Merck: Grant/Research Support|Pfizer: Advisor/Consultant|Pfizer: Grant/Research Support

